# Polyketide-Derived Secondary Metabolites from a Dothideomycetes Fungus, *Pseudopalawania siamensis* gen. et sp. nov., (Muyocopronales) with Antimicrobial and Cytotoxic Activities

**DOI:** 10.3390/biom10040569

**Published:** 2020-04-08

**Authors:** Ausana Mapook, Allan Patrick G. Macabeo, Benjarong Thongbai, Kevin D. Hyde, Marc Stadler

**Affiliations:** 1 Institute of Plant Health, Zhongkai University of Agriculture and Engineering, Haizhu District, Guangzhou 510225, China; phung.ausana@gmail.com; 2 Center of Excellence in Fungal Research, Mae Fah Luang University, Chiang Rai 57100, Thailand; 3 Department Microbial Drugs, Helmholtz Centre for Infection Research, and German Centre for Infection Research (DZIF), partner site Hannover-Braunschweig, Inhoffenstrasse 7, 38124 Brunswick, Germany; agmacabeo@ust.edu.ph (A.P.G.M.); benjarong.zine.thongbai@gmail.com (B.T.); 4 Laboratory for Organic Reactivity, Discovery and Synthesis (LORDS), Research Center for the Natural and Applied Sciences, University of Santo Tomas, 1015 Manila, Philippines

**Keywords:** ascomycota, biological activity, multi-gene phylogenetic, new genus, new species, taxonomy, structure elucidation

## Abstract

*Pseudopalawania siamensisgen*. et *sp*. *nov*., from northern Thailand, is introduced based on multi-gene analyses and morphological comparison. An isolate was fermented in yeast malt culture broth and explored for its secondary metabolite production. Chromatographic purification of the crude ethyl acetate (broth) extract yielded four tetrahydroxanthones comprised of a new heterodimeric bistetrahydroxanthone, pseudopalawanone (**1**), two known dimeric derivatives, 4,4′-secalonic acid D (**2**) and penicillixanthone A (**3**), the corresponding monomeric tetrahydroxanthone paecilin B (**4**), and the known benzophenone, cephalanone F (**5**). Compounds **1**–**3** showed potent inhibitory activity against Gram-positive bacteria. Compounds **2** and **3** were inhibitory against *Bacillus subtilis* with minimum inhibitory concentrations (MIC) of 1.0 and 4.2 μg/mL, respectively. Only compound **2** showed activity against *Mycobacterium smegmatis*. In addition, the dimeric compounds **1**–**3** also showed moderate cytotoxic effects on HeLa and mouse fibroblast cell lines, which makes them less attractive as candidates for development of selectively acting antibiotics.

## 1. Introduction

Fungi are potentially known as a promising source of bioactive compounds for drug discovery [1]. Mushrooms and other Basidiomycota, in particular, are widely used in traditional Chinese medicines and have been shown to provide beneficial activities against cancer and other ailments [2,3], but even the microfungi have various other potential benefits [4]. Dothideomycetes (Ascomycota) is a large and diverse class comprising of mostly microfungi. New species are constantly being discovered from this group and could be promising sources of novel bioactive compounds [5,6,7]. A few contemporary studies in Thailand have been focusing on saprobic fungi in Dothideomycetes as a source for finding novel bioactive compounds. For example, a novel Thai Dothideomycete, *Pseudobambusicola thailandica*, has yielded six new compounds with nematicidal and antimicrobial activity [8]. A new abscisic acid derivative with anti-biofilm activity against *Staphylococcus aureus* was isolated from cultures of a *Roussoella* sp. inhabiting *Clematis subumbellata* in northern Thailand [9], while *Sparticola junci*, another new Thai dothideomycete, yielded seven new spirodioxynaphthalenes with antimicrobial and cytotoxic activities [10]. Recently some phenalenones from another new Thai *Pseudolophiostoma* species were found to selectively inhibit α-glucosidase and lipase [11]. In spite of these recent discoveries, the study of bioactive compounds from Thai and other tropical Dothideomycetes is still in the initial stages of research.

In this study, we provide morphological descriptions and illustrations of a new Dothideomycetes fungus *Pseudopalawania siamensis*, collected from *Caryota* sp. (Arecaceae) in northern Thailand, based on multi-gene analyses and morphological comparison to confirm the current taxonomic placement of the fungus. In addition, we studied the new fungus for the production of bioactive compounds because its extracts showed significant antimicrobial activities in a preliminary screening. Thus, we here report the first secondary metabolites from this species, including their isolation, structure elucidation, and biological activity.

## 2. Materials and Methods

### 2.1. Sample Collection, Specimen Examination and Isolation of Fungi

Fresh material was collected from Nan Province, Thailand, in 2016. Fungal micromorphology was examined using a Motic, (Hongkong, China) SMZ 168 Series microscope. The appearance of ascomata on substrate was captured using a (stereo microscope fitted with an AxioCam ERC 5S camera (Carl Zeiss GmbH, Jena, Germany). Sections of ascomata were made by free hand. Fungal material was mounted in water and photographed with a Nikon (Bangkok, Thailand) ECLIPSE Ni compound microscope fitted with a Canon (Singapore) EOS 600D digital camera. Fungal photoplate was processed with Adobe Photoshop CS6 version 13.1.2 (Adobe Systems, CA, USA). All microscopic characters were measured using Tarosoft Image Frame Work program (IFW) version 0.97 (Nonthaburi, Thailand). Single spore isolations were obtained using the methods of Chomnunti et al. [12]. Germinating ascospores were transferred to a new malt extract agar (MEA) media and incubated at room temperature (25 °C) in the dark. Fungal cultures were used for molecular study and secondary metabolite production. The specimens and living cultures are deposited in the Herbarium of Mae Fah Luang University (Herb. MFLU) and Culture collection Mae Fah Luang University (MFLUCC), Chiang Rai, Thailand. Nomenclature and taxonomic information were deposited in MycoBank [13].

### 2.2. DNA Extraction, PCR Amplification and Sequencing

The genomic DNA from the fungal mycelium was extracted by using the ZR Soil Microbe DNA MiniPrep kit (Zymo Research, Irvine, CA, USA) following the manufacturer’s instructions. DNA amplifications were performed by polymerase chain reaction (PCR). The partial large subunit nuclear rDNA (LSU) was amplified with primer pairs LROR and LR5 [14]. The internal transcribed spacer (ITS) was amplified by using primer pairs ITS5 and ITS4 [15]. The partial small subunit nuclear rDNA (SSU) was amplified with primer pairs NS1 and NS4 [15]. The translation elongation factor 1-alpha gene (TEF1) was amplified by using primers EF1-983F and EF1-2218R [16]. The partial gene encoding for the second largest RNA polymerase subunit (RPB2) was amplified by using primers fRPB2-5F and fRPB2- 7cR [17]. Methods for PCR amplification and sequencing were carried out according to previously described procedures [18,19].

### 2.3. Phylogenetic Analysis

The closest matched taxa were determined through nucleotide BLAST searches online in GenBank (http://www.ncbi.nlm.nih.gov/). Combined LSU: 28S large subunit of the nrRNA gene; ITS: internal transcribed spacer regions 1 and 2 including 5.8S nrRNA gene; SSU: 18S small subunit of the nrRNA gene; TEF1: partial translation elongation factor 1-α gene; and RPB2: partial RNA polymerase II second largest subunit gene sequence data from representative closest relatives to our strains were selected following Hongsanan et al. [20], Crous et al. [21], Hernández-Restrepo et al. [22], and Mapook et al. [23,24], to confirm the phylogenetic placement of our new strains. The phylogenetic analysis based on maximum likelihood (ML) and Bayesian inference (BI) were following the methodology as described in Mapook et al. [23,24]. The sequences used for analyses with accession numbers are given in Table 1. Phylogram generated from ML analysis was drawn using FigTree v. 1.4.2 [25] and edited by Microsoft Office PowerPoint 2013. The new nucleotide sequence data are deposited in GenBank.

### 2.4. General Information of Chromatography and Spectral Methods

Specific optical rotations ([α]_D_) were measured using a Perkin-Elmer (Überlingen, Germany) 241 polarimeter in a 100 × 2 mm cell at 22 °C. ECD spectra were recorded on a J-815 spectropolarimeter (JASCO, Pfungstadt, Germany). UV spectra were obtained on a Shimadzu (Duisburg, Germany) UV-Vis spectrophotometer UV-2450 with 1 cm quartz cells. IR spectra were measured with a Nicolet Spectrum 100 FT-IR spectrometer (Perkin-Elmer, Waltham, MA, USA). Nuclear magnetic resonance (NMR) spectra were recorded on a Bruker 700 MHz Avance III spectrometer with a 5 mm TXI cryoprobe (^1^H 700 MHz, ^13^C 175 MHz) and a Bruker 500 MHz Avance III spectrometer with a BBFO (plus) SmartProbe (^1^H 500 MHz, ^13^C 125 MHz). In all cases, spectra were acquired at 25 °C (unless otherwise specified) in solvents as specified in the text, with referencing to residual ^1^H or ^13^C signals in the deuterated solvents (CDCl_3_ or MeOH-*d*_4_). HPLC-DAD/MS analysis was conducted using an amaZon Speed ETD ion trap mass spectrometer (Bruker Daltonics, Bremen, Germany). HR-ESI mass spectra was measured using an Agilent 1200 series HPLC-UV system (column 2.1 × 50 mm, 1.7 μm, C18 Waters Acquity UPLC BEH) combined with an maXis (Bruker) ESI-TOF-MS instrument The mobile phase was composed of H_2_O + 0.1% formic acid (solvent A) and acetonitrile + 0.1% formic acid (solvent B), with the following gradient: 5% solvent B for 0.5 min with a flow rate of 0.6 mL/min, increasing to 100% solvent B in 19.5 min and then maintaining 100% solvent B for 5 min. UV/Vis detection at 200–600 nm. Chemicals and solvents were obtained from AppliChem GmbH, Avantor Performance Materials, Carl Roth GmbH & Co. KG (Karlsruhe, Germany) and Merck KGaA (Darmstadt, Germany) in analytical and HPLC grade.

### 2.5. Fermentation and Extraction

Five mycelial plugs from actively growing colonies on malt extract agar (MEA) media (malt extract 20 g/L, D-glucose 20 g/L, peptone 6 g/L, pH 6.3) were cut using a sterile cork borer (0.7 × 0.7 cm^2^) and placed into a sterilized 500 mL Erlenmeyer flask containing 200 mL of liquid yeast malt (YM) medium (malt extract 10 g/L, D-glucose 4 g/L, yeast extract 4 g/L, pH 6.3). These seed cultures were incubated on a rotary shaker (140 rpm) at 23 °C in the dark for nine days. Ten milliliters of the seed culture were added into 25 × 500 mL sterile Erlenmeyer flasks with 200 mL of YM medium and incubated on a rotary shaker for 14 days. The extraction was conducted 3 days after glucose depletion as monitored by the glucose strip test using Bayer Harnzuckerstreifen, (Bayer, Leverkusen, Germany). Fungal mycelium and supernatant were separated by using vacuum filtration. The supernatant was mixed with 3% Amberlite XAD-16N adsorber resin (Sigma-Aldrich, Deisenhofen, Germany) and stirred for 1 h and filtrated to remove the culture broth. The XAD resin was eluted three times with an equal volume of ethyl acetate. The mycelia were extracted twice with an equal volume of acetone in an ultrasonic bath for 30 min and the combined extracts were passed through a filter, then dissolved in water/ethyl acetate. The aqueous phase (lower) was discarded while the organic phase (upper) was filtered through anhydrous sodium sulfate (Na_2_SO_4_) for water removal and then evaporated to dryness. This procedure yielded 1580 mg mycelial crude extract and 769 mg of supernatant crude extract. The mycelial extract contained mainly fatty acids and ergosterol derivatives and showed only weak bioactivity. It was therefore not further processed. The supernatant extract contained the majority of the active components and was therefore subjected to preparative isolation of its active ingredients.

### 2.6. Isolation of Compounds ***1***–***5***

**Figure 1:**
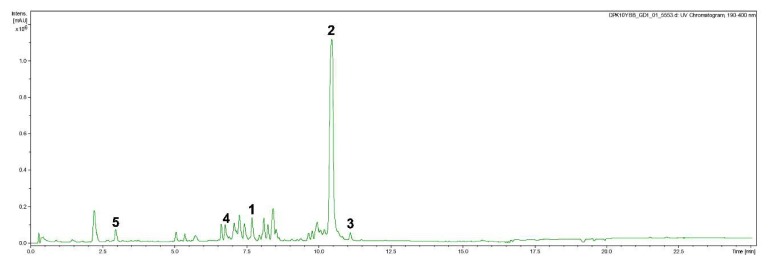
HPLC-(DAD)-UV chromatogram of the crude ethyl acetate extract of the culture filtrate of *Pseudopalawania siamensis* (MFLUCC 17-1476).

The supernatant crude extract was dissolved in methanol and initially fractionated on preparative HPLC manufactured by Gilson (Middleton, Wi, USA), comprised of a GX-271 Liquid Handler, a 172 DAD, a 305 and 306 pump, with 50SC Piston Pump Head. A Phenomenex (Torrance, Ca., USA) Gemini 10u C_18_ 110Å column (250 × 21.20 mm, 10 μm) was used as a stationary phase. The mobile phase was composed of deionised water (Milli-Q, Millipore, Schwalbach, Germany) with 0.05% of trifluoroacetic acid (TFA) as a solvent A and acetonitrile (ACN) HPLC grade with 0.05% TFA as a solvent B. The fractionation proceeded with the following gradient: linear gradient of 10% solvent B for 5 min with a flow rate of 35 mL/min, followed by 10% to 100 % solvent B for 30 min, and 100% solvent B for 10 min. The UV detection was carried out at 210, 254 and 350 nm. Final five compounds were purified from initially 16 fractions (Figure 1). Compound **1** (pseudopalawanone; 5.51 mg) eluted at *t*_R_ = 7.8 min from fraction 12, compound **2** (4,4′-secalonic acid D; 5.48 mg) eluted at *t*_R_ = 10.5 min from fraction 15, compound **4** (paecilin B; 1.08 mg) eluted at *t*_R_ = 6.9 min from fraction 4, and compound **5** (cephalanone F; 1.52 mg) eluted at *t*_R_ = 3.0 min from fraction 3, while compound **3** (penicillixanthone A; 0.86 mg) eluted at *t*_R_ = 11.3 min was resulted from the purification of fraction 16 (4.12 mg) on a VarioPrep Nucleodur 100-10 C_18_ ec column (150 *×* 40 mm, 7 µm; Macherey-Nagel, Düren, Germany) using the following gradients: linear gradient of 30% solvent B for 5 min with a flow rate of 15 mL/min, followed by 30% to 100 % solvent B for 20 min, and 100% solvent B for 10 min.

### 2.7. Spectral Data

#### Pseudopalawanone (**1**)

Pale yellowish gum. [a]^25^_D_ = +30.0 (*c* 1.0, MeOH). ^1^H NMR (500 MHz, CDCl_3_): see Table 2; ^13^C NMR (125 MHz, CDCl_3_): see Table 2. HR-ESIMS *m/z* 641.1492 ([M + H]^+^, calcd for C_31_H_29_O_15_, 641.1501).

### 2.8. Antimicrobial Activity and Cytotoxicity Assays

Minimum inhibitory concentrations (MIC) of compounds **1**–**5** were determined against various fungal and bacterial strains by using a 96-well serial dilution technique according to previously described procedures [92,93]. The tested organisms with results are given in Table 3 and Table 4. Gentamicin, kanamycin, nystatin, and oxytetracycline were used as positive controls against tested organisms. In vitro cytotoxicity (IC_50_) of compounds **1**–**5** were determined using the MTT assay according to previously described procedures [26,27] against the mouse fibroblast cell line (L929) and the human HeLa (KB-3-1) cell line. Epothilone B and methanol were used as positive and negative control, respectively.

## 3. Results and Discussion

### 3.1. Phylogenetic Analysis

**Figure 2:**
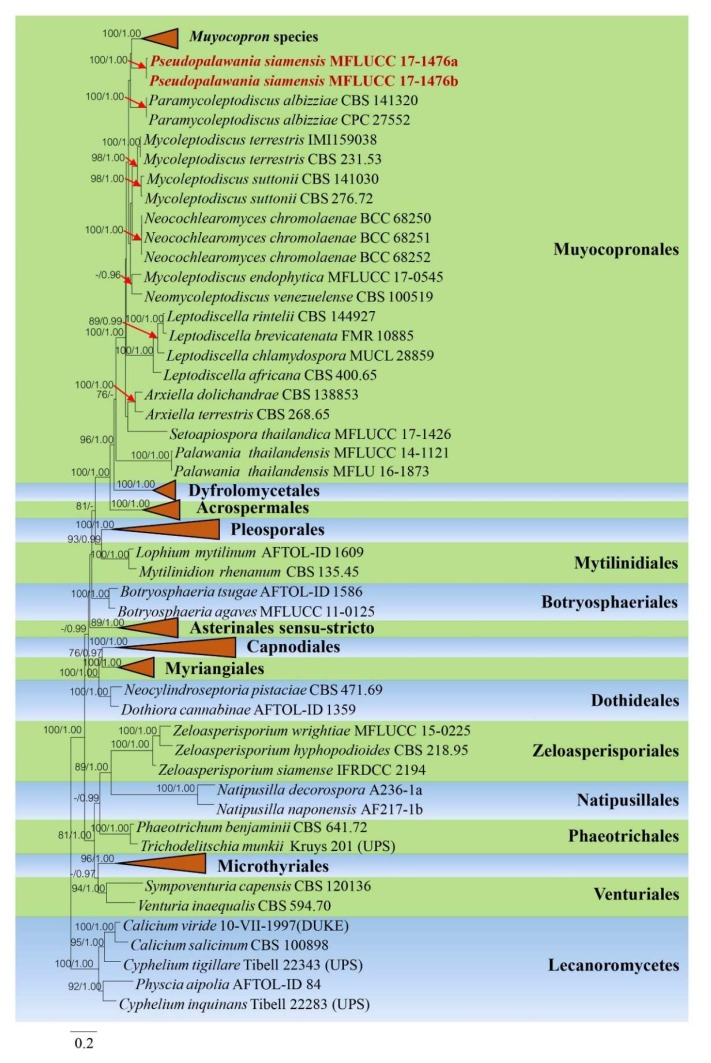
Phylogram generated from maximum likelihood analysis based on combined dataset of LSU, SSU, RPB2, ITS and TEF sequence data. Bootstrap support values for maximum likelihood (ML) equal to or greater than 60% and Bayesian posterior probabilities (PP) equal to or greater than 0.90 are given above the nodes. Newly generated sequences are in dark red bold. The tree is rooted with Lecanoromycetes. Small red arrows point towards the bootstrap values of the clades representing genera of the order Muyocopronales, while some other monophyletic clades that represent monophyletic clades have been collapses (indicated by red triangles).

The combined dataset of LSU, SSU, RPB2, ITS and TEF sequence data including our new strains were analyzed by maximum likelihood (ML) and Bayesian analyses. The combined sequence alignment is comprised of 155 taxa (6131 characters with gaps), which include representative strains from Lecanoromycetes as outgroup taxa. A best scoring RAxML tree with a final likelihood value of -91,669.392085 is presented in Figure 2. The matrix had 3930 distinct alignment patterns, with 59.36% of undetermined characters or gaps. Estimated base frequencies were as follows: A = 0.242975, C = 0.253394, G = 0.277569, T = 0.226061; substitution rates: AC = 1.292278, AG = 3.020191, AT = 1.589713, CG = 1.197479, CT = 6.661698, GT = 1.000000; gamma distribution shape parameter α = 0.357175. In a BLASTn search of NCBI GenBank, the closest matches of the ITS sequence of *Pseudopalawania siamensis* (MFLUCC 17-1476, ex-holotype) is *Muyocopron geniculatum* with 81.40% (MK487737) similarity, respectively, was strain CBS. 721.95, the closest matches of the SSU sequence with 98.90% similarity, was *Neocochlearomyces chromolaenae* (strain BCC 68250, NG_065766), the closest matches of the TEF sequence with 95.17% similarity, was *Neomycoleptodiscus venezuelense* (strain CBS 100519, MK495978). The phylogram generated from maximum likelihood analysis (Figure 2) shows that our new strains clustered within Dothideomycetes and form a distinct lineage in the Muyocopronales, even though the clade is lacking bootstrap support.

### 3.2. Taxonomy

#### 3.2.1. *Pseudopalawania* Mapook and K.D. Hyde, gen. nov.

*Mycobank number*: MB834934.

*Etymology*: The generic epithet refers to the similarity to *Palawania*.

*Saprobic* on dead rachis of Arecaceae. **Sexual morph**: *Ascomata* superficial, solitary or scattered, sub-carbonaceous to carbonaceous, appearing as circular, flattened, dark brown to black spots, covering the host, without a subiculum, with a poorly developed basal layer and an irregular margin. *Ostioles* central. *Peridium* comprising dark brown or black to reddish-brown cells of *textura epidermoidea* to *textura angularis*. *Hamathecium* cylindrical to filiform, septate, hyaline, branching pseudoparaphyses. *Asci* eight-spored, bitunicate, fissitunicate, cylindric-clavate, straight or slightly curved, with an ocular chamber observed clearly when immature. *Ascospores* overlapping, 2–3-seriate, broadly fusiform to inequilateral, pointed ends, hyaline, 1-septate, constricted at the septum, guttulate when immature, surrounded by hyaline and thin layers of gelatinous sheath, observed clearly when mounted in Indian ink. **Asexual morph**: Undetermined.

*Type species: **Pseudopalawania siamensis*** Mapook and K.D. Hyde

#### 3.2.2. ***Pseudopalawania siamensis*** Mapook and K.D. Hyde, sp. nov.

**Figure 3:**
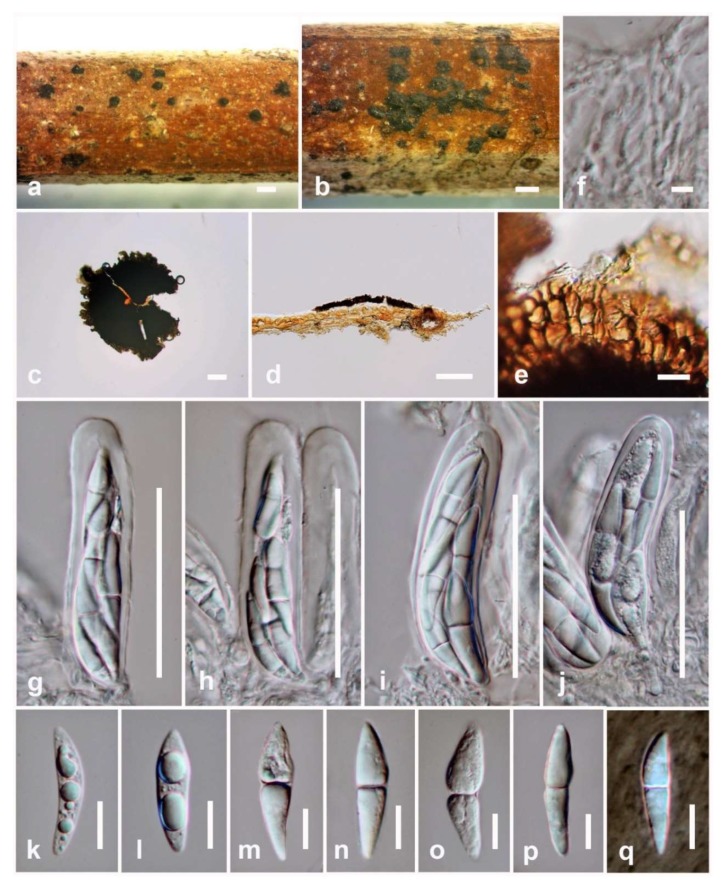
***Pseudopalawania siamensis*** (**holotype**) (**a**,**b**) Appearance of ascomata on substrate. (**c**) Squash mounts showing ascomata. (**d**) Section of ascoma. (**e**) Peridium. (**f**) Pseudoparaphyses. (**g**–**j**) asci. (**k**–**p**) Ascospores. (**q**) Ascospores in Indian ink. **Scale bars**: a, b = 500 µm, c, d = 100 µm, g–j = 50 µm, e, k–q = 10 µm, f = 5 µm.

*Mycobank number*: MB834935; Figure 3

*Etymology*: Named after the country from where the fungus was collected, using the former name of Siam.

*Saprobic* on dead rachis of *Caryota* sp. **Sexual morph**: *Ascomata* 29–40 µm high × 270–290(–315) µm diam. ($\bar{x}$ = 32.5 × 292 µm, n = 5), superficial, solitary or scattered, sub-carbonaceous to carbonaceous, appearing as circular, flattened, dark brown to black spots, covering the host, without a subiculum, with a poorly developed basal layer and an irregular margin. *Ostioles* central. *Peridium* 10–20 µm wide, comprising dark brown or black to reddish-brown cells of *textura epidermoidea* to *textura angularis*. *Hamathecium* comprising 1–2.5 µm wide, cylindrical to filiform, septate, hyaline, branching pseudoparaphyses. *Asci* 65–85 × 15–21 µm ($\bar{x}$ = 75 × 18 µm, n = 10), eight-spored, bitunicate, fissitunicate, cylindric-clavate, straight or slightly curved, with an ocular chamber observed clearly when immature. *Ascospores* 25–37 × 5–11 µm ($\bar{x}$ = 29 × 7 µm, n = 20), overlapping, 2–3-seriate, broadly fusiform to inequilateral, pointed ends, hyaline, 1-septate, constricted at the septum, guttulate when immature, surrounded by hyaline and thin layers of gelatinous sheath, observed clearly when mounted in Indian ink. **Asexual morph**: Undetermined.

**Culture characteristics**: Ascospores germinating on MEA within 24 hrs. at room temperature and germ tubes produced from the apex. Colonies on MEA circular, slightly raised, filamentous, mycelium white at the surface and initially creamy-white to pale brown in reverse, becoming dark brown from the centre of the colony with creamy-white at the margin.

**Pre-screening for antimicrobial activity**: *Pseudopalawania siamensis* (MFLUCC 17-1476) showed antimicrobial activity against *B. subtilis* with a 16 mm inhibition zone and against *M. plumbeus* with a 17 mm inhibition zone, observable as full inhibition, when compared to the positive control (26 mm and 17 mm, respectively), but no inhibition of *E. coli*.

**Material examined**: THAILAND, Nan Province, on dead rachis of *Caryota* sp. (Arecaceae), 23 September 2016, A. Mapook (MFLU 20-0353, **holotype**); ex-type culture MFLUCC 17-1476.

*Notes*: *Pseudopalawania* is similar to *Palawania* in its superficial and flattened ascomata, with hyaline, 1-septate ascospores, but differs in its peridium wall patterns, shape of asci (cylindric-clavate vs. inequilateral to ovoid) with an ocular chamber and shape of ascospores (broadly fusiform to inequilateral vs. oblong to broadly fusiform) with a thin layer of gelatinous sheath. The gelatinous sheath in *Palawania* is thicker [24]. *Pseudopalawania* is also similar to *Muyocopron* in its superficial, flattened ascomata with similar peridium wall patterns, and asci with an ocular chamber; but differs in its sub-carbonaceous to carbonaceous ascomata, shape of asci and ascospores with surrounded by hyaline gelatinous sheath, 1-septate, while *Muyocopron* have coriaceous ascomata, aseptate ascospores with granular appearance and without gelatinous sheath [23]. In addition, the genus was compared with genera in Microthyriaceae of which no DNA sequence data are available, but the holotype specimens were re-examined in previous studies with morphological descriptions and illustrations [94,95,96,97,98,99], and neither of them matched our new fungus. Therefore, we introduce *Pseudopalawania* as a new genus with a new species *P. siamensis* from Thailand. The fungus is placed in Muyocopronaceae (Muyocopronales) with evidence from morphology and phylogeny.

### 3.3. Structure Elucidation of the New Compound

**Figure 4:**
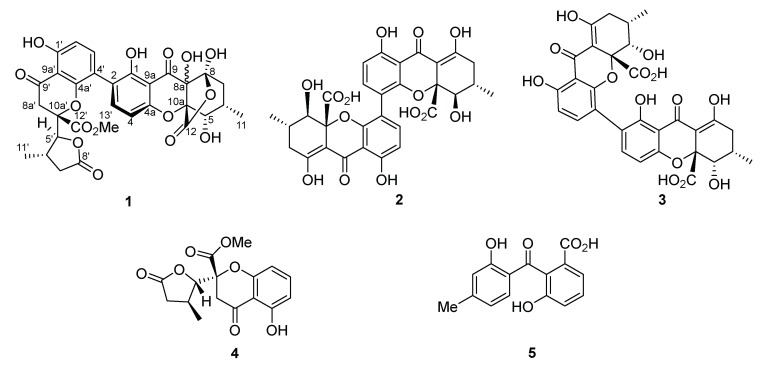
Secondary metabolites from *Pseudopalawania siamensis*.

HPLC chromatographic fractionation of the crude ethyl acetate extract from the yeast malt (YM 6.3) broth of *Pseudopalawania siamensis* resulted in the isolation of a new heterodimeric bistetrahydroxanthone, pseudopalawanone (**1**) together with three known tetrahydroxanthones, 4,4′-secalonic acid D (**2**) [100], penicillixanthone A (**3**) [101], paecilin B (**4**) [102] and the benzophenone, cephalanone F (**5**) [103] (Figure 4).

**Figure 5:**
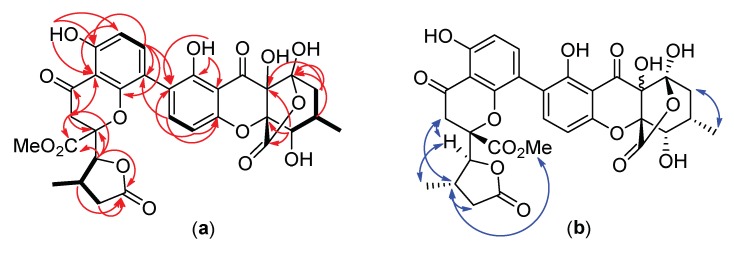
COSY (bold bonds), HMBC (red arrows) (**a**), and ROESY (blue arrows) (**b**) correlations in pseudopalawanone (**1**).

**Figure 6:**
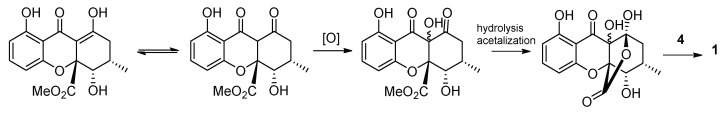
Plausible biogenetic pathway towards pseudopalawanone (**1**).

Pseudopalawanone (**1**) was obtained as optically active, pale yellow gum. The IR spectrum showed the presence of hydroxyl groups (3387 cm^−1^), carbonyl functionalities (1787, 1741 cm^−1^) and aromatic residues (1648, 1622 cm^−1^) while the UV spectrum was indicative of absorptions due to chromanone units [102,104]. The molecular formula C_31_H_28_O_15_, indicating eighteen double bond equivalents, was established by HR-ESIMS based on its protonated pseudomolecular ion peak ([M + H]^+^) at *m/z* 641.1492. Observation of two sets of signals in the NMR spectra (Figures S1 and S2) and careful comparison of the ^1^H and ^13^C NMR spectroscopic data of **1** (Table 2) with those of **2**–**4** immediately revealed **1** to be an asymmetric dimer of an unfamiliar highly oxygenated tetrahydroxanthone subunit and 7-deoxyblennolide D [102]. Thus, the gross structure of the latter fragment along with its connection to 7-deoxyblennolide D was established through analysis of 1D and 2D NMR spectroscopic data and will be the subject of the following discussions. The ^13^C and HSQC-DEPT edited spectra (Figure S3) showed the presence of fifteen resonances comprised of a ketone (δ*_C_* 194.9), a carboxyl group of an ester functionality (δ*_C_* 176.6), a hemiacetal carbon (δ*_C_* 108.9), four quaternary aromatic carbons (δ*_C_* 106.8, 117.6, 158.3, 160.1), two aromatic methine carbons (δ*_C_* 108.3, 143.8), two aliphatic quaternary carbons (δ*_C_* 73.6, 84.7), two methine carbons (δ*_C_* 30.4, 74.1), a methylene carbon (δ*_C_* 33.8) and a methyl group (δ*_C_* 14.9). The ^1^H and COSY NMR spectrum (Figure S4) revealed two ortho-coupled aromatic protons (^3^*J* = 8.6 Hz) for H–3 (δ*_H_* 7.82) and H–4 (δ*_H_* 6.77), and a seven-proton spin system comprised of H–5 (δ*_H_* 4.44) – H–6 (δ*_H_* 2.23) (H_3_–11) (δ*_H_* 1.20) – H_2_–7 (δ*_H_* 2.12, 2.36). A C–2 substituted 1-hydroxychromanone unit was elucidated on the basis of HMBC correlations of chelated 1-OH (δ*_H_* 11.35) with C–1 (δ*_C_* 160.1), C–2 (δ*_C_* 117.6) and C–9a (δ*_C_* 106.8) and of H–4 (δ*_H_* 6.77) with C–2 and C–4a (δ*_C_* 158.3). The remaining portion of the molecule was constructed through HMBC correlations of H–6 (δ*_H_* 2.13) and H–11 (δ*_H_* 1.20) with C–8 (δ*_C_* 108.9), of H–5 (δ*_H_* 4.44) with C–8a (δ*_C_* 73.6), C–10a (δ*_C_* 84.7) and C–12 (δ*_C_* 176.6), and of H_2_–7 (δ*_H_* 2.12, 2.36) with C–8 and C–8a. The chemical shifts assigned for C–8 and C–12 were ascribed to hemiacetal and *γ*-lactone moieties, respectively, by using a combination of 2D NMR experiments (Figure 5). The lactone ester was plausibly attached to C–8 forming a *γ*-hydroxylactone subunit of a [3.2.1] bicyclic structure. The remaining 17 mass units was attributed to a hydroxyl group attached to the *〈*−carbon (C–8a) of the chromanone substructure. This unusual tetrahydroxanthone motif could putatively originate presumably from *α* hydroxylation of the keto form of blennolide A, followed by nucleophilic attack of the hydrolyzed C–12 methyl ester (Figure 6). The relative configurations of C–5 and C–6 were readily established to be similar with blennolide A by the coupling constant (^3^*J*_5,6_ = 4.0 Hz) and the chemical shifts as 5S*, 6S* while that of C–10a was assigned R* based on the observed positive n-π* CD transition at around 331 nm [104]. The chirality of C–8a cannot be established using available methods due to its remoteness to most protons in the molecule.

The linkage between the chromanone subunit and the *©*−lactone in the 7-deoxyblennolide D monomer was indicated by the HMBC correlation of H–5′ (δ*_H_* 4.38) with C–10a′ (δ*_C_* 84.8) and C–12′ (δ*_C_* 168.5). The C–5′S* and C–6′S* relative configurations in the lactone moiety were established by coupling constant analysis (^3^*J*_5,6_ = 2.5 Hz) depicting a pseudodiaxial orientation for H–5′/H–6′ and the NOE (Figures S6 and S7) noted between H–5′ and H–8a’*a* (δ*_H_* 3.14), H–8a′*b* (δ*_H_* 2.98) and H–6′ (δ*_H_* 2.65), and that of H–6′ and H_3_–13 (δ*_H_* 3.80) [102]. The spatial arrangements in ring C were similar to 7-deoxyblennolide D corroborated by NOE correlations between H–5′, H_3_–11′ (δ*_H_* 1.16) and H–7′b (δ*_H_* 1.99). Finally, the relative configuration of C-10a′ may be tentatively assigned as S* on the basis of negative π*-π* transitions below 330 nm and positive n-π* transitions at 346 nm in the ECD spectrum (Figure S9) of **1** [104]. The overall relative configuration of the blennolide-type tetrahydroxanthone substructure is 5S*, 6S*, and 10aS* thus, structurally similar to 7-deoxyblennolide D. 

The planar structure of **1** was established by connecting the two monomers through the linkage of C–2 (δ*_C_* = 117.6) of the oxidized secalonic acid subunit and C–4′ (δ*_C_* 114.0) of 7-deoxyblennolide D evidenced by the diagnostic HMBC correlations of H–3 (δ*_H_* 7.82) to C–4′ and H–3′ (δ*_H_* 7.54) to C–2. The axial configuration of C-2/C-4′ was assigned as *P* based on the CD spectrum of **1** which showed a positive first Cotton effect (225 nm, De = −6.41) and a negative second cotton effect (250 nm, De = +3.15). Thus, compound **1** was given the trivial name pseudopalawanone. To establish unambiguously its relative and absolute configurations especially C–8a in the blennolide A substructure and C–10a’ in the 7-deoxyblennolide D substructure, we suggest additional experiments such as asymmetric total synthesis, derivatization with heavy atom/s followed by single crystal x-ray diffraction and/or further ECD-TDDFT measurements and calculations.

### 3.4. Biological Activity of Compounds ***1***–***5***

The polyketides **1**–**5** were evaluated for their antimicrobial activity against selected microorganisms (Table 3) and cytotoxicity against two mammalian cell lines, HeLa cells KB3.1 and mouse fibroblast cell line L929 (Table 4). The starting concentration for antimicrobial assay and cytotoxicity assay were 66.7 and 300 µg/mL, respectively and the substances were dissolved in MeOH (1 mg/mL). MeOH was used as the negative control and showed no activity against the tested organisms and mammalian cell lines. Results were expressed as MIC or minimum inhibitory concentration (μg/mL) and IC_50_ or half maximal inhibitory concentration (μM) (Table 3 and Table 4). The known compounds **4** and **5** showed neither antimicrobial nor cytotoxic activities. The dimeric tetrahydroxanthone 4,4′-secalonic acid D (**2**) showed inhibition against the pathogenic fungus *Candida albicans* while penicillixanthone A (**3**) inhibited *Mucor hiemalis* with activities comparable to the positive drug control nystatin. Prominent activities were observed for compounds **2** and **3** against *Bacillus subtilis* with MIC values of 1.0 and 4.2 μg/mL, respectively. Compound **2** also showed inhibitory activity against all Gram-positive bacteria (*Bacillus subtilis*, *Micrococcus luteus*, *Mycobacterium smegmatis*, and *Staphylococcus aureus*), while compounds **1** and **3** also showed inhibitory activity against the Gram-positive bacterium, *Mycobacterium smegmatis*. In general, only the dimeric tetrahydroxanthones **1**–**3** exhibited activity against fungi and bacteria with the secalonic acid-bearing derivatives **2** and **3** exhibiting better antimicrobial profile. However, the dimeric compounds **1**–**3** also showed moderate cytotoxic activities against two mammalian cell lines (Table 4). These inhibitory concentrations for cytotoxic activities are given traditionally in molar concentrations, but if they are calculated in µg/mL, the IC_50_ values would be equivalent to a range of 2–25 µg/mL (i.e., the same or only slightly higher activity range as compared to the MIC). This observation precludes the potential use of these metabolites as candidates for the development of antibiotics, because their selectivity indices are far too low. In addition, the fact that they are broadly active against both, prokaryotic and eukaryotic test organisms suggests that they may address multiple targets and are therefore less suitable for development of any drug.

Some information on these and chemically related compounds is even available from the literature. Compound **2** (4,4′-secalonic acid D; 4,4′-SAD) is a regioisomeric structure to SAD with 2,2′-biarylic connectivity, belonging to the secalonic acid family. This compound class has long been known to have non-selective antimicrobial and other biological activities [100,101,102,103,104,105,106]. The compound 4,4′-SAD (2) itself was recently reported to have low toxicity with “potent” antitumor activity against several cancer cell lines through cell proliferation inhibition and apoptosis induction [100]. However, when compared to the precursor for a marketed drug, epothilone, which we used as a positive control in our standard cytotoxicity assays (Table 4), the activities of all the metabolites from *Pseudopalawania siamensis* are much weaker. Promising candidate compounds for anticancer therapy should have at least activities in the 100 nM range such assays. Penicillixanthone A (**3**) was also already shown to possess moderate antibacterial activity against four tested bacterial strains (*M. luteus*, *Pseudoalteromonas nigrifaciens*, *E. coli* and *B. subtilis* [100], and its moderate cytotoxic effects on MDA-MB-435 human melanoma cells and SW620 human colorectal adenocarcinoma cell lines had been previously reported [101]. Furthermore, compound **3** was previously isolated from the marine-derived fungus *Aspergillus fumigatus*, and was reported to exhibit anti-HIV-1 activities by inhibiting CCR5-tropic HIV-1 and CXCR4-tropic HIV-1 infection [103]. These data also point toward non-selective effects of this metabolite in biological systems. 

## 4. Conclusions

The current study showed that new genera and species of tropical fungi can still yield numerous new and interesting secondary metabolites. Even though the preliminary characterization of the metabolites **1**–**5** indicates that they act non-selectively in biological systems, their further evaluation could result in the discovery of additional, more specific biological effects. In any case, it is worthwhile to further explore tropical fungi whose cultures result from taxonomic and biodiversity studies for the production of secondary metabolites and other potentially beneficial properties [107].

## Figures and Tables

**Table 1:** Taxa used in this study and their GenBank accession numbers. New sequences generated in the present study are in bold.

Taxa	Strain No. ^1^	GenBank Accession Numbers ^2^					References
		LSU	SSU	RPB2	ITS	TEF	
***Acrospermum adeanum***	M133	EU940104	EU940031	EU940320	EU940180	-	Stenroos et al. [26]
***Acrospermum compressum***	M151	EU940084	EU940012	EU940301	EU940161	-	Stenroos et al. [26]
***Acrospermum gramineum***	M152	EU940085	EU940013	EU940302	EU940162	-	Stenroos et al. [26]
***Alternaria alternata***	KFRD-18	KX609781	KX609769	-	KX346897	KY094931	Li et al. [27]
***Alternariaster bidentis***	CBS 134021	KC609341	-	KC609347	KC609333	-	Alves et al. [28]
***Antennariella placitae***	CBS:124785	GQ303299	-	-	MH863403	-	Cheewangkoon et al. [29]
***Arxiella dolichandrae***	CBS 138853	KP004477	-	-	KP004449	-	Crous et al. [30]
***Arxiella terrestris***	CBS 268.65	MH870201	-	-	MH858565	-	Vu et al. [31]
***Asterina fuchsiae***	TH590	GU586216	GU586210	-	-	-	Hofmann et al. [32]
***Asterina phenacis***	TH589	GU586217	GU586211	-	-	-	Hofmann et al. [32]
***Bambusicola massarinia***	MFLUCC 11-0389	JX442037	JX442041	KU940169	JX442033	-	Dai et al. [33]
***Bambusicola splendida***	MFLUCC 11-0439	JX442038	JX442042	-	JX442034	-	Dai et al. [33]
***Botryosphaeria agaves***	MFLUCC 11-0125	JX646808	JX646825	-	JX646791	JX646856	Liu et al. [34]
***Botryosphaeria tsugae***	AFTOL-ID 1586	DQ767655	-	DQ767644	-	DQ677914	Schoch et al. [35]
***Calicium salicinum***	CBS 100898	KF157982	KF157970	KF157998	-	-	Beimforde et al. [36]
***Calicium viride***	10-VII-1997 (DUKE)	AF356670	AF356669	AY641031	-	-	Lutzoni et al. [37]
***Camarosporium quaternatum***	CBS 483.95	GU301806	GU296141	GU357761	KY929149	GU349044	Schoch et al. [38]
***Capnodium salicinum***	AFTOL-ID 937	DQ678050	DQ677997	-	-	DQ677889	Schoch et al. [37]
***Caryospora minima***	-	EU196550	EU196551	-	-	-	Cai and Hyde [39]
***Chaetothyriothecium elegans***	CPC 21375	KF268420	-	-	-	-	Hongsanan et al. [40]
***Corynespora cassiicola***	CBS 100822	GU301808	GU296144	GU371742	-	GU349052	Schoch et al. [38]
***Corynespora smithii***	CABI 5649b	GU323201	-	GU371783	-	GU349018	Schoch et al. [38]
***Cucurbitaria berberidis***	MFLUCC 11-0387	KC506796	KC506800	-	-	-	Hyde et al. [41]
***Cyphelium inquinans***	Tibell 22283 (UPS)	AY453639	U86695	-	AY450584	-	Tibell [42]
***Cyphelium tigillare***	Tibell 22343 (UPS)	AY453641	AF241545	-	AY452497	-	Tibell [42]
***Cystocoleus ebeneus***	L161	EU048578	EU048571	-	-	-	Muggia et al. [43]
***Didymella exigua***	CBS 183.55	JX681089	EU754056	GU371764	MH857436	KR184187	Verkley et al. [44]
***Didymosphaeria rubi-ulmifolii***	MFLUCC 14-0023	KJ436586	KJ436588	-	-	-	Ariyawansa et al. [45]
***Dothiora cannabinae***	AFTOL ID 1359	DQ470984	DQ479933	DQ470936	-	DQ471107	Spatafora et al. [46]
***Dyfrolomyces phetchaburiensis***	MFLUCC 15-0951	MF615402	MF615403	-	-	-	Hyde et al. [47]
***Dyfrolomyces rhizophorae***	BCC15481	-	KF160009	-	-	-	Pang et al. [48]
***Dyfrolomyces rhizophorae***	JK 5456A	GU479799	-	-	-	GU479860	Suetrong et al. [49]
***Dyfrolomyces thailandica***	MFLU 16-1173	KX611366	KX611367	-	-	-	Hyde et al. [50]
***Dyfrolomyces thamplaensis***	MFLUCC 15-0635	KX925435	KX925436	-	-	KY814763	Zhang et al. [51]
***Dyfrolomyces tiomanensis***	NTOU3636	KC692156	KC692155	-	-	KC692157	Pang et al. [48]
***Elsinoe fawcettii***	CPC 18535	JN940382	JN940559	-	KX887207	KX886853	Schoch et al. [52]
***Elsinoe verbenae***	CPC 18561	JN940391	JN940562	-	KX887298	KX886942	Schoch et al. [53]
***Extremus antarcticus***	CCFEE 5312	KF310020	-	KF310086	KF309979	-	Egidi et al. [54]
***Gonatophragmium triuniae***	CBS 138901	KP004479	-	-	KP004451	-	Crous et al. [30]
***Helicascus nypae***	BCC 36751	GU479788	GU479754	GU479826	-	GU479854	Suetrong et al. [49]
***Julella avicenniae***	BCC 20173	GU371822	GU371830	GU371786	-	GU371815	Schoch et al. [38]
***Karschia cezannei***	Cezanne-Eichler B26	KP456152	-	-	-	-	Ertz and Diederich [55]
***Katumotoa bambusicola***	KT 1517a	AB524595	AB524454	AB539095	NR_154103	AB539108	Tanaka et al. [56]
***Labrocarpon canariense***	Ertz 16907 (BR)	KP456157	-	-	-	-	Ertz and Diederich [55]
***Lentithecium fluviatile***	CBS 123090	FJ795450	FJ795492	FJ795467	-	-	Zhang et al. [57]
***Leptodiscella africana***	CBS 400.65	MH870275	-	-	MH858635	-	Vu et al. [31]
***Leptodiscella brevicatenata***	FMR 10885	FR821311	-	-	FR821312	-	Madrid et al. [58]
***Leptodiscella chlamydospora***	MUCL 28859	FN869567	-	-	FR745398	-	Madrid et al. [58]
***Leptodiscella rintelii***	CBS 144927	LR025181	-	-	LR025180	-	Papendorf [52]
***Leptosphaeria doliolum***	MFLUCC 15-1875	KT454719	KT454734	-	KT454727	-	Ariyawansa et al. [59]
***Leptosphaerulina australis***	CBS 317.83	EU754166	GU296160	GU371790	MH861604	GU349070	de Gruyter et al. [60]
***Leptoxyphium cacuminum***	MFLUCC10-0049	JN832602	JN832587	-	-	-	Chomnunti et al. [61]
***Lophiotrema nucula***	CBS 627 86	GU301837	GU296167	GU371792	LC194497	GU349073	Schoch et al. [38]
***Lophium mytilinum***	AFTOL-ID 1609	DQ678081	DQ678030	DQ677979	-	DQ677926	Schoch et al. [35]
***Massarina bambusina***	H 4321	AB807536	AB797246	-	LC014578	AB808511	Tanaka et al. [56]
***Massarina eburnea***	CBS 473.64	GU301840	GU296170	GU371732	-	GU349040	Schoch et al. [38]
***Melanomma pulvis-pyrius***	CBS 371 75	GU301845	FJ201989	GU371798	-	GU349019	Schoch et al. [38]
***Melaspileopsis*** **cf. *diplasiospora***	Ertz 16247 (BR)	KP456164	-	-	-	-	Ertz and Diederich [55]
***Melomastia maolanensis***	GZCC 16-0102	KY111905	KY111906	-	-	KY814762	Zhang et al. [51]
***Microsphaeropsis olivacea***	CBS 233 77	GU237988	-	KT389643	MH861055	-	Aveskamp et al. [62]
***Microthyrium buxicola***	MFLUCC 15-0213	KT306552	KT306550	-	-	-	Ariyawansa et al. [63]
***Microthyrium microscopicum***	CBS 115976	GU301846	GU296175	GU371734	-	GU349042	Schoch et al. [44]
***Multiseptospora thailandica***	MFLUCC 11-0183	KP744490	KP753955	-	KP744447	KU705657	Liu et al. [64]
***Murispora rubicunda***	IFRD 2017	FJ795507	GU456308	-	-	GU456289	Zhang et al. [57]
***Muyocopron alcornii***	BRIP 43897	MK487708	-	MK492712	MK487735	MK495956	Hernández-Restrepo et al. [22]
***Muyocopron atromaculans***	MUCL 34983	MK487709	-	MK492713	MK487736	MK495957	Hernández-Restrepo et al. [22]
***Muyocopron castanopsis***	MFLUCC 10-0042	-	JQ036225	-	-	-	Mapook et al. [23]
***Muyocopron castanopsis***	MFLUCC 14-1108	KU726965	KU726968	KY225778	**MT137784**	**MT136753**	Mapook et al. [23]
***Muyocopron chromolaenae***	MFLUCC 17-1513	MT137876	MT137881	MT136761	MT137777	MT136756	Mapook et al. [24]
***Muyocopron chromolaenicola***	MFLUCC 17-1470	MT137877	MT137882	-	MT137778	MT136757	Mapook et al. [24]
***Muyocopron coloratum***	CBS 720.95	MK487710	-	MK492714	NR_160197	MK495958	Hernández-Restrepo et al. [22]
***Muyocopron dipterocarpi***	MFLUCC 14-1103	KU726966	KU726969	KY225779	**MT137785**	**MT136754**	Mapook et al. [23]
***Muyocopron dipterocarpi***	MFLUCC 17-0075	MH986833	MH986829	-	MH986837	-	Senwanna et al. [65]
***Muyocopron dipterocarpi***	MFLUCC 17-0354	MH986834	MH986830	-	MH986838	-	Senwanna et al. [65]
***Muyocopron dipterocarpi***	MFLUCC 17-0356	MH986835	MH986831	-	MH986839	-	Senwanna et al. [66]
***Muyocopron dipterocarpi***	MFLUCC 18-0470	MK348001	MK347890	-	MK347783	-	Jayasiri et al. [67]
***Muyocopron garethjonesii***	MFLU 16-2664	KY070274	KY070275	-	-	-	Tibpromma et al. [68]
***Muyocopron geniculatum***	CBS 721.95	MK487711	-	MK492715	MK487737	MK495959	Hernández-Restrepo et al. [22]
***Muyocopron heveae***	MFLUCC 17-0066	MH986832	MH986828	-	MH986836	-	Senwanna et al. [66]
***Muyocopron laterale***	CBS 141029	MK487712	-	MK492716	MK487738	MK495960	Hernández-Restrepo et al. [22]
***Muyocopron laterale***	IMI 324533	MK487713	-	MK492717	MK487739	MK495961	Hernández-Restrepo et al. [22]
***Muyocopron laterale***	CBS 719.95	MK487714	-	MK492718	MK487740	MK495962	Hernández-Restrepo et al. [22]
***Muyocopron laterale***	CBS 141033	MK487715	-	MK492719	MK487741	MK495963	Hernández-Restrepo et al. [22]
***Muyocopron laterale***	URM 7802	MK487716	-	MK492720	MK487742	MK495964	Hernández-Restrepo et al. [22]
***Muyocopron laterale***	URM 7801	MK487717	-	MK492721	MK487743	-	Hernández-Restrepo et al. [22]
***Muyocopron laterale***	CBS 127677	MK487718	-	MK492722	MK487744	MK495965	Hernández-Restrepo et al. [22]
***Muyocopron laterale***	CBS 145310	MK487719	-	MK492723	MK487745	MK495966	Hernández-Restrepo et al. [22]
***Muyocopron laterale***	CBS 145315	MK487720	-	MK492724	MK487746	MK495967	Hernández-Restrepo et al. [22]
***Muyocopron laterale***	CBS 145313	MK487721	-	MK492725	MK487747	MK495968	Hernández-Restrepo et al. [22]
***Muyocopron laterale***	CBS 145309	MK487722	-	MK492726	MK487748	MK495969	Hernández-Restrepo et al. [22]
***Muyocopron laterale***	CBS 145314	MK487723	-	MK492727	MK487749	MK495970	Hernández-Restrepo et al. [22]
***Muyocopron laterale***	CBS 145311	MK487724	-	MK492728	MK487750	-	Hernández-Restrepo et al. [22]
***Muyocopron laterale***	CBS 145312	MK487725	-	MK492729	MK487751	MK495971	Hernández-Restrepo et al. [22]
***Muyocopron laterale***	CBS 145316	MK487726	-	MK492730	MK487752	MK495972	Hernández-Restrepo et al. [22]
***Muyocopron laterale***	FMR13797	MK874616	-	MK875802	MK874615	MK875803	Hernández-Restrepo et al. [22]
***Muyocopron lithocarpi***	MFLUCC 10-0041	JQ036230	JQ036226	-	-	-	Mapook et al. [23]
***Muyocopron lithocarpi***	MFLUCC 14-1106	KU726967	KU726970	KY225780	**MT137786**	**MT136755**	Mapook et al. [23]
***Muyocopron lithocarpi***	MFLU 18-2087	MK347930	MK347821	-	MK347716	-	Jayasiri et al. [66]
***Muyocopron lithocarpi***	MFLU 18-2088	MK347931	MK347822	-	MK347717	-	Jayasiri et al. [66]
***Muyocopron lithocarpi***	MFLUCC 16-0962	MK348034	MK347923	-	-	-	Jayasiri et al. [66]
***Muyocopron lithocarpi***	MFLUCC 17-1465	MT137878	MT137883	-	MT137779	MT136758	Mapook et al. [24]
***Muyocopron lithocarpi***	MFLUCC 17-1466	MT137879	MT137884	-	MT137780	MT136759	Mapook et al. [24]
***Muyocopron lithocarpi***	MFLUCC 17-1500	MT137880	MT137885	MT136762	MT137781	MT136760	Mapook et al. [24]
***Muyocopron zamiae***	CBS 203.71	MK487727	-	MK492731	-	MK495973	Hernández-Restrepo et al. [22]
***Mycoleptodiscus endophytica***	MFLUCC 17-0545	MG646946	MG646978	-	MG646961	MG646985	Tibpromma et al. [69]
***Mycoleptodiscus suttonii***	CBS 276.72	MK487728	-	MK492732	MK487753	MK495974	Hernández-Restrepo et al. [22]
***Mycoleptodiscus suttonii***	CBS 141030	MK487729	-	MK492733	-	MK495975	Hernández-Restrepo et al. [22]
***Mycoleptodiscus terrestris***	CBS 231.53	MK487730	-	MK492734	MK487754	MK495976	Hernández-Restrepo et al. [22]
***Mycoleptodiscus terrestris***	IMI 159038	MK487731	-	MK492735	MK487755	MK495977	Hernández-Restrepo et al. [22]
***Myriangium duriaei***	CBS 260.36	NG_027579	AF242266	KT216528	MH855793	-	Schoch et al. [35]
***Myriangium hispanicum***	CBS 247.33	GU301854	GU296180	GU371744	MH855426	GU349055	Schoch et al. [38]
***Mytilinidion rhenanum***	CBS 135.34	FJ161175	FJ161136	FJ161115	-	FJ161092	Boehm et al. [70]
***Natipusilla decorospora***	AF236 1a	HM196369	HM196376	-	-	-	Ferrer et al. [71]
***Natipusilla naponensis***	AF217 1a	HM196371	HM196378	-	-	-	Ferrer et al. [71]
***Neocochlearomyces chromolaenae***	BCC 68250	MK047514	MK047552	-	MK047464	MK047573	Crous et al. [21]
***Neocochlearomyces chromolaenae***	BCC 68251	MK047515	MK047553	-	MK047465	MK047574	Crous et al. [21]
***Neocochlearomyces chromolaenae***	BCC 68252	MK047516	MK047554	-	MK047466	MK047575	Crous et al. [21]
***Neocylindroseptoria pistaciae***	CBS 471.69	KF251656	-	KF252161	KF251152	KF253112	Quaedvlieg et al. [65]
***Neomycoleptodiscus venezuelense***	CBS 100519	MK487732	-	MK492736	MK487756	MK495978	Hernández-Restrepo et al. [22]
***Palawania thailandensis***	MFLUCC 14-1121	KY086493	KY086495	KY086496	**MT137787**	-	Mapook et al. [24]
***Palawania thailandensis***	MFLU 16-1871	KY086494	-	-	**MT137788**	-	Mapook et al. [24]
***Paramycoleptodiscus albizziae***	CPC 27552	MH878220	-	-	-	-	Vu et. al. [31]
***Paramycoleptodiscus albizziae***	CBS 141320	KX228330	-	MK492737	KX228279	MK495979	Crous et. al. [72]
***Phaeodimeriella cissampeli***	MFLU 16-0558	KU746806	KU746808	KU746810	-	KU746812	Mapook et. al. [73]
***Phaeodimeriella dilleniae***	MFLU 14-0013	KU746805	KU746807	KU746809	-	KU746811	Mapook et. al. [73]
***Phaeotrichum benjaminii***	CBS 541.72	AY004340	AY016348	GU357788	MH860561	DQ677892	Lumbsch et. al. [74]
***Physcia aipolia***	AFTOL-ID 84	DQ782904.1	DQ782876	DQ782862	DQ782836	DQ782892	James et. al. [75]
***Piedraia hortae***	CBS 480.64	GU214466	-	KF902289	GU214647	-	Crous et. al. [76]
***Platystomum crataegi***	MFLUCC 14-0925	KT026109	KT026113	-	NG_063580	KT026121	Thambugala et. al. [77]
***Pleomassaria siparia***	AFTOL-ID 1600	DQ678078	DQ678027	DQ677976	-	DQ677923	Schoch et. al. [35]
***Pleospora herbarum***	IT 956	KP334709	KP334729	KP334733	KP334719	KP334731	Ariyawansa et. al. [78]
***Preussia funiculata***	CBS 659.74	GU301864	GU296187	GU371799	-	GU349032	Schoch et. al. [38]
***Pseudomassariosphaeria bromicola***	IT-1333	KT305994	KT305996	-	KT305998	KT305999	Ariyawansa et. al. [63]
***Pseudopalawania siamensis***	**MFLUCC 17-1476a**	-	**MT137789**	-	**MT137782**	**MT136752**	**This study**
***Pseudopalawania siamensis***	**MFLUCC 17-1476b**	-	**MT137790**	-	**MT137783**	-	**This study**
***Pseudostrickeria muriformis***	MFLUCC 13-0764	KT934254	KT934258	-	-	KT934262	Tian et. al. [79]
***Pseudovirgaria grisea***	CPC 19134	JF957614	-	-	JF957609	-	Braun et. al. [80]
***Pseudovirgaria hyperparasitica***	CPC 10753	EU041824	-	-	EU041767	-	Arzanlou et. al. [81]
***Ramularia endophylla***	CBS 113265	KF251833	-	KP894673	KF251220	-	Verkley et. al. [82]
***Rasutoria pseudotsugae***	rapssd	EF114704	EF114729	-	EF114687	-	Winton et al. [83]
***Rasutoria tsugae***	ratstk	EF114705	EF114730	GU371809	EF114688	-	Winton et al. [83]
***Salsuginea ramicola***	KT 2597.1	GU479800	GU479768	GU479833	-	GU479861	Suetrong et al. [49]
***Schizothyrium pomi***	CBS 406.61	EF134949	-	KF902384	-	-	Batzer et al. [84]
***Setoapiospora thailandica***	MFLUCC 17-1426	MN638847	MN638851	-	MN638862	MN648731	Hyde et al. [85]
***Stictographa lentiginosa***	Ertz 17570 (BR)	KP456170	-	-	-	-	Ertz and Diederich [55]
***Sympoventuria capensis***	CBS 120136	KF156104	KF156094	-	KF156039	-	Samerpitak et al. [86]
***Teratosphaeria fibrillosa***	CBS 121707	GU323213	GU296199	GU357767	MH863138	KF903305	Schoch et al. [38]
***Trichodelitschia munkii***	Kruys 201 (UPS)	DQ384096	DQ384070	-	-	-	Kruys et al. [87]
***Tumidispora*** ***shoreae***	MFLUCC 14-0574	KT314074	KT314076	-	-	-	Ariyawansa et al. [63]
***Uwebraunia commune***	NC132C1d	-	-	KT216546	-	-	Ismail et al. [88]
***Venturia inaequalis***	CBS 594.70	GU301879	GU296205	GU357757	KF156040	GU349022	Schoch et al. [38]
***Xenolophium applanatum***	CBS 123127	GU456330	GU456313	GU456355	-	GU456270	Zhang et al. [89]
***Zeloasperisporium hyphopodioides***	CBS 218.95	EU035442	-	-	-	-	Crous et al. [90]
***Zeloasperisporium siamense***	IFRDCC 2194	JQ036228	JQ036223	-	-	-	Mapook et. al. [73]
***Zeloasperisporium wrightiae***	MFLUCC 15-0225	KT387737	KT387738	-	-	-	Hongsanan et al. [91]

^1^ AFTOL-ID: Assembling the Fungal Tree of Life; BCC: BIOTEC Culture Collection; BRIP: Biosecurity Queensland Plant Pathology Herbarium, Brisbane, Australia; CBS: Westerdijk Fungal Biodiversity Institute, Utrecht, The Netherlands; CCFEE: Culture Collection of Fungi from Extreme Environments, The University of Tuscia; CPC: Culture collection of Pedro Crous, the Netherlands; FMR: Facultad de Medicina, Reus, Tarragona, Spain; GZCC: Guizhou Culture Collection; IFRDCC = International Fungal Research and Development Centre Culture Collection, China; IMI: The International Mycological Institute Culture Collections; JK: J. Kohlmeyer; MFLU: the Herbarium of Mae Fah Luang University; MFLUCC: Mae Fah Luang University Culture Collection, Chiang Rai, Thailand; MUCL: Belgian Coordinated Collections of Microorganisms; URM: Universidade Federal de Pernambuco. ^2^ LSU: 28S large subunit of the nrRNA gene; SSU: 18S small subunit of the nrRNA gene; RPB2: partial RNA polymerase II second largest subunit gene; ITS: internal transcribed spacer regions 1 and 2 including 5.8S nrRNA gene; TEF1: partial translation elongation factor 1-α gene.

**Table 2:** NMR spectroscopic data for pseudopalawanone (**1**).

No.	*δ_H_*, m, *J* (Hz)	*δ_C_*, m	No.	*δ_H_*, m, *J* (Hz)	*δ_C_*, m
1	-	160.1, C	1′	-	161.8, C
2	-	117.6, C	2′	6.66, d (8.7)	110.4, CH
3	7.82, d (8.6)	143.8, CH	3′	7.54, d (8.7)	141.2, CH
4	6.77, d (8.6)	108.3, CH	4′	-	114.0, C
4a	-	158.3, C	4a′	-	155.6, C
5	4.44, d (4.0)	74.1, CH	5′	4.38, d (2.5)	88.1, CH
6	2.13, m	30.4, CH	6′	2.65, m	29.9, CH
7*a*	2.36, dd (15.9, 13.6)	33.8, CH_2_	7′*a*	2.18, m	35.8, CH_2_
*b*	2.12, m		*b*	1.99, dd (18.3, 3.1)	
8	-	108.9, C	8′	-	176.5, C
8a	-	73.6, C	8a′*a*	3.14, d (16.9)	39.6, CH_2_
			*b*	2.98, d (16.9)	
9	-	194.9, C	9′	-	193.6, C
9a	-	106.8, C	9a′	-	107.6, C
10a	-	84.7, C	10a′	-	84.8, C
11	1.20, d (6.5)	14.9, CH_3_	11′	1.16, d (7.2)	20.9, CH_3_
12	-	176.6, C	12′	-	168.5, C
13	-	-	13′	3.80, s	53.7, CH_3_
1-OH	11.35, s	-	1′-OH	11.51, s	-

**Table 3:** Antimicrobial activity of compounds **1**–**5**.

Tested Organisms	Strain No.	Minimum Inhibitory Concentration (MIC) [μg/mL]					
		Compounds					Positive Control *
		1	2	3	4	5	
***Fungi***							
*Candida albicans*	DSM 1665	-	66.7	-	-	-	66.7 (20 µL N)
*Cryptococcus neoformans*	DSM 15466	-	-	-	-	-	66.7 (20 µL N)
*Mucor hiemalis*	DSM 6766	-	-	66.7	-	-	66.7 (20 µL N)
*Pichia anomala*	DSM 6766	-	-	-	-	-	66.7 (20 µL N)
*Rhodoturula glutinis*	DSM 10134	-	-	-	-	-	16.7 (20 µL N)
*Schizosaccharomyces pombe*	DSM 70572	-	-	-	-	-	33.3 (20 µL N)
***Bacteria***							
*Bacillus subtilis*	DSM 10	66.7	1.0	4.2	-	-	8.3 (20 µL O)
*Chromobacterium violaceum*	DSM 30191	-	-	-	-	-	1.7 (2 µL O)
*Escherichia coli*	DSM 1116	-	-	-	-	-	3.3 (2 µL O)
*Micrococcus luteus*	DSM 1790	66.7	8.3	33.3	-	-	0.4 (2 µL O)
*Mycobacterium smegmatis*	ATCC 700084	-	66.7	-	-	-	3.3 (2 µL K)
*Pseudomonas aeruginosa*	PA14	-	-	-	-	-	0.8 (2 µL G)
*Staphylococcus aureus*	DSM 346	66.7	4.2	33.3	-	-	0.2 (2 µL O)

* Positive drug controls: K = kanamycin, N = nystatin, O = oxytetracycline hydrochloride. (-): no inhibition. The starting concentration was 66.7 µg/mL.

**Table 4:** Cytotoxic activity of compounds **1**–**5**.

Cell Lines	IC_50_ (µM)					
	Compounds					Epothilone B
	1	2	3	4	5	
*HeLa cells KB3.1*	29.7	3.9	17.2	-	-	8.9 × 10^−5^
*Mouse fibroblast L929*	50.0	14.1	-	-	-	1.8 × 10^−3^

The *in vitro* cytotoxicity test of polyketides **1**–**5** was conducted against two mammalian cell lines, with epothilone B as positive control. Starting concentration for cytotoxicity assay was 66 μg/mL, substances were dissolved in MeOH (1 mg/mL). MeOH was used as negative control and showed no activity against the tested mammalian cell lines. Results were expressed as IC_50_: half maximal inhibitory concentration (µM). (-): no inhibition.

## Supplementary Materials

The following are available online at https://www.mdpi.com/2218-273X/10/4/569/s1, Figure S1: 1H NMR spectrum (CDCl3, 700 MHz) of pseudopalawanone (**1**). Figure S2: 13C NMR spectrum (CDCl3, 175 MHz) of pseudopalawanone (**1**). Figure S3: HSQC-DEPT spectrum of pseudopalawanone (**1**). Figure S4: COSY spectrum of pseudopalawanone (**1**). Figure S5: HMBC spectrum of pseudopalawanone (**1**). Figure S6: ROESY spectrum of pseudopalawanone (**1**). Figure S7: NOESY spectrum of pseudopalawanone (**1**). Figure S8: LC-HRESIMS spectrum of pseudopalawanone (**1**). Figure S9: ECD spectrum of pseudopalawanone (**1**).
