# Supplementary material for: Polyketide-Derived Secondary Metabolites from a Dothideomycetes Fungus, Pseudopalawania siamensis gen. et sp. nov., (Muyocopronales) with Antimicrobial and Cytotoxic Activities
Source: Biomolecules. 2020 Apr 8;10(4):569. doi: 10.3390/biom10040569 (PMC7226469; doi:10.3390/biom10040569)
Supplement: Supplementary file 1 [file biomolecules-10-00569-s001.pdf]

SUPPLEMENTARY MATERIAL FOR

Polyketide-derived secondary metabolites from a Dothideomycetes fungus, Pseudopalawania siamense gen. et sp. nov., (Muyocopronales) with antimicrobial and cytotoxic activities

Ausana Mapook,[1] Allan Patrick G. Macabeo,[2,3] Benjarong Thongbai,[2] Kevin D. Hyde,[1] and Marc Stadler[2,*]

[1]Center of Excellence in Fungal Research, Mae Fah Luang University, Chiang Rai, 57100 Thailand

[2]Department of Microbial Drugs, Helmholtz Centre for Infection Research and German Centre for Infection Research (DZIF), partner site Hannover/Braunschweig, Inhoffenstrasse 7, 38124, Braunschweig, Germany

[3]Laboratory for Organic Reactivity, Discovery and Synthesis (LORDS), Research Center for the Natural and Applied Sciences, University of Santo Tomas, 1015 Manila, Philippines

[1]These authors contributed equally on this work.

LIST OF SUPPORTING INFORMATION

	Page
**S1.** ^{1}H NMR spectrum ($CDCl_3$, 700 MHz) of pseudopalawanone (**1**)	3
**S2.** ^{13}C NMR spectrum ($CDCl_3$, 175 MHz) of pseudopalawanone (**1**)	4
**S3.** HSQC-DEPT spectrum of pseudopalawanone A (**1**)	5
**S4.** COSY spectrum of pseudopalawanone (**1**)	6
**S5.** HMBC spectrum of pseudopalawanone (**1**)	7
**S6.** ROESY spectrum of pseudopalawanone (**1**)	8
**S7.** NOESY spectrum of pseudopalawanone (**1**)	9
**S8.** LC-HR-ESIMS spectrum of pseudopalawanone (**1**)	10
**S9.** CD spectrum (MeOH) of pseudopalawanone (**1**)	10

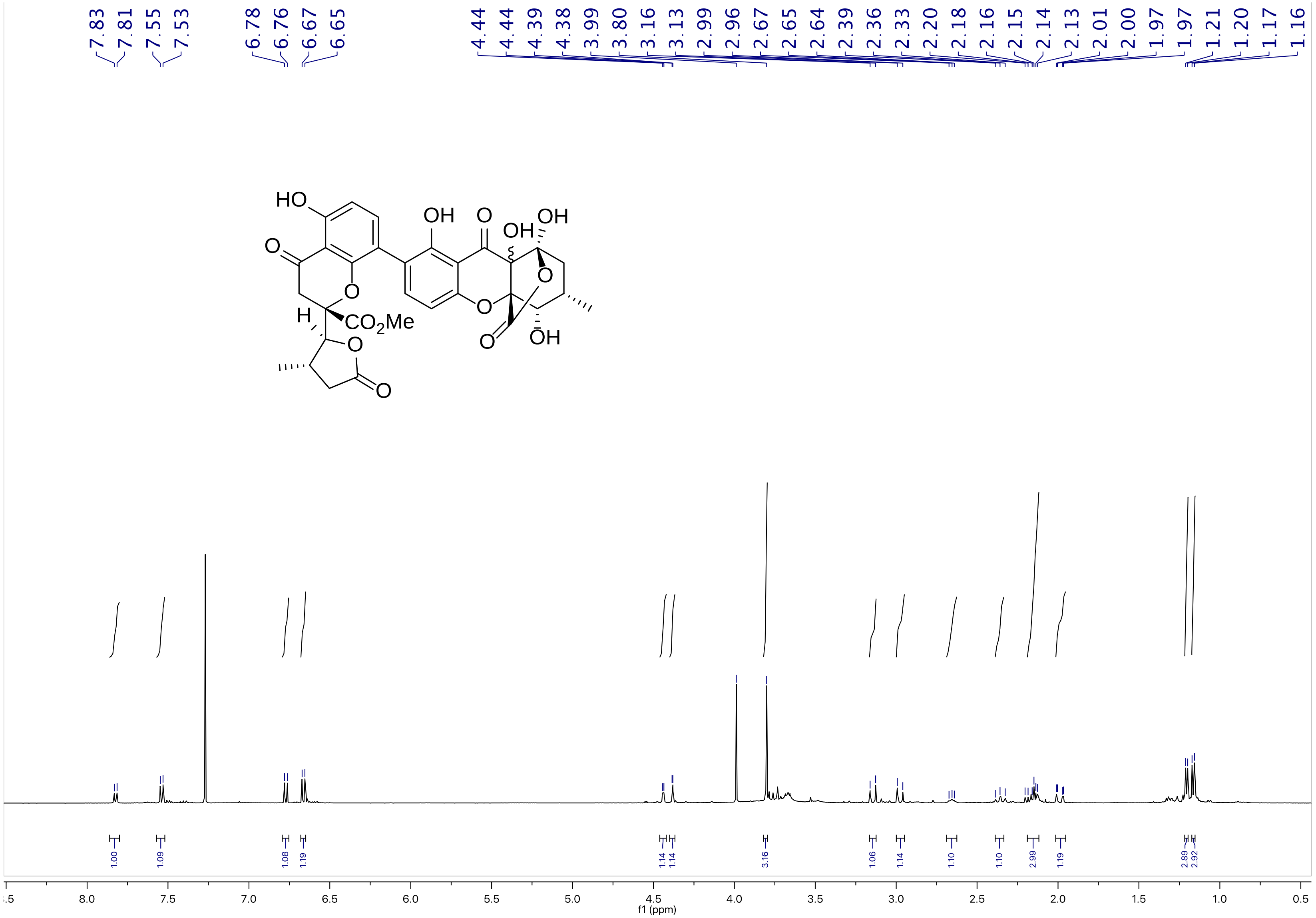

Figure S1. ^{1}H NMR spectrum ($CDCl_3$, 700 MHz) of pseudopalawanone A (**1**).

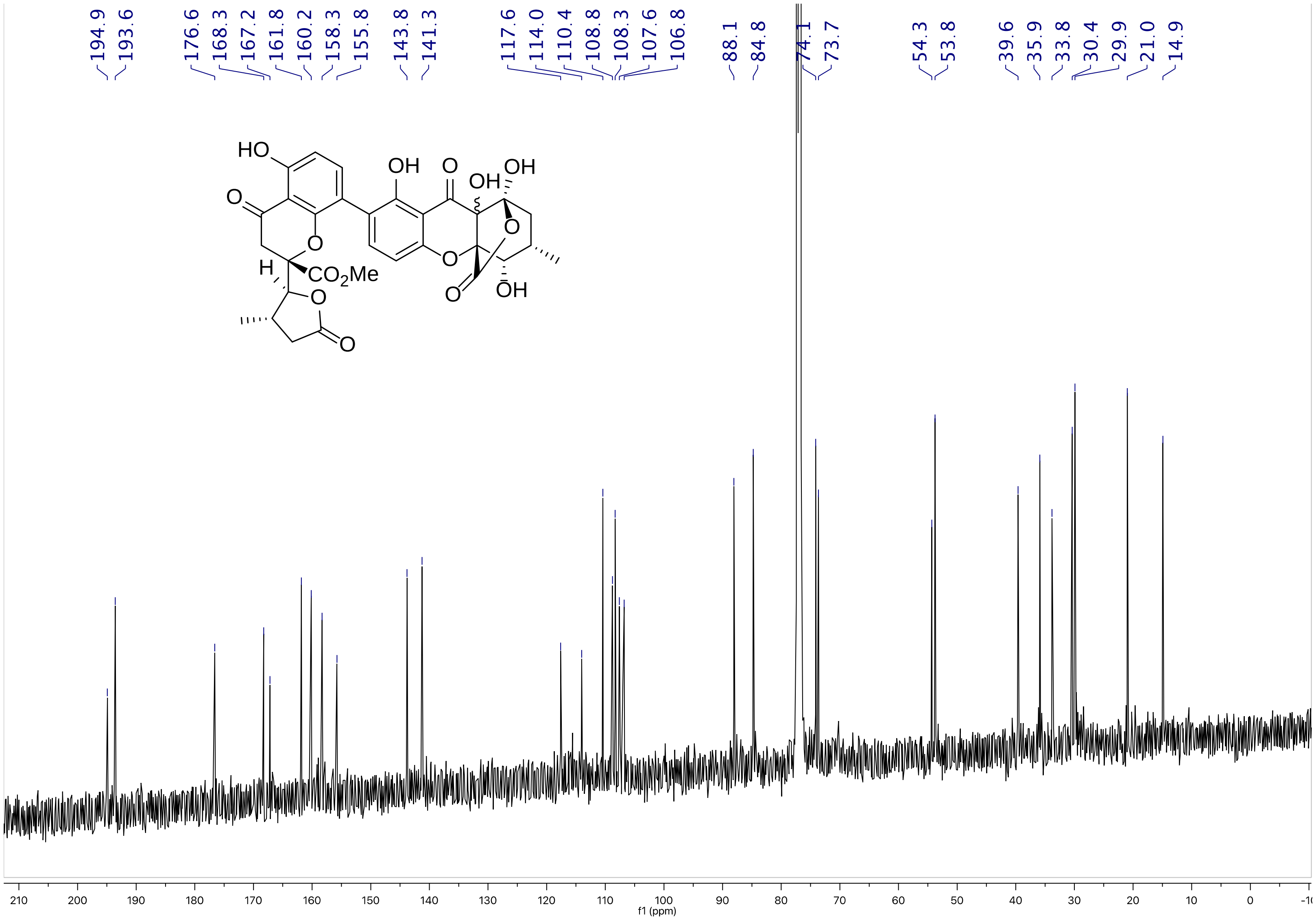

Figure S2. ^{13}C NMR spectrum ($CDCl_3$, 175 MHz) of pseudopalawanone A (**1**).

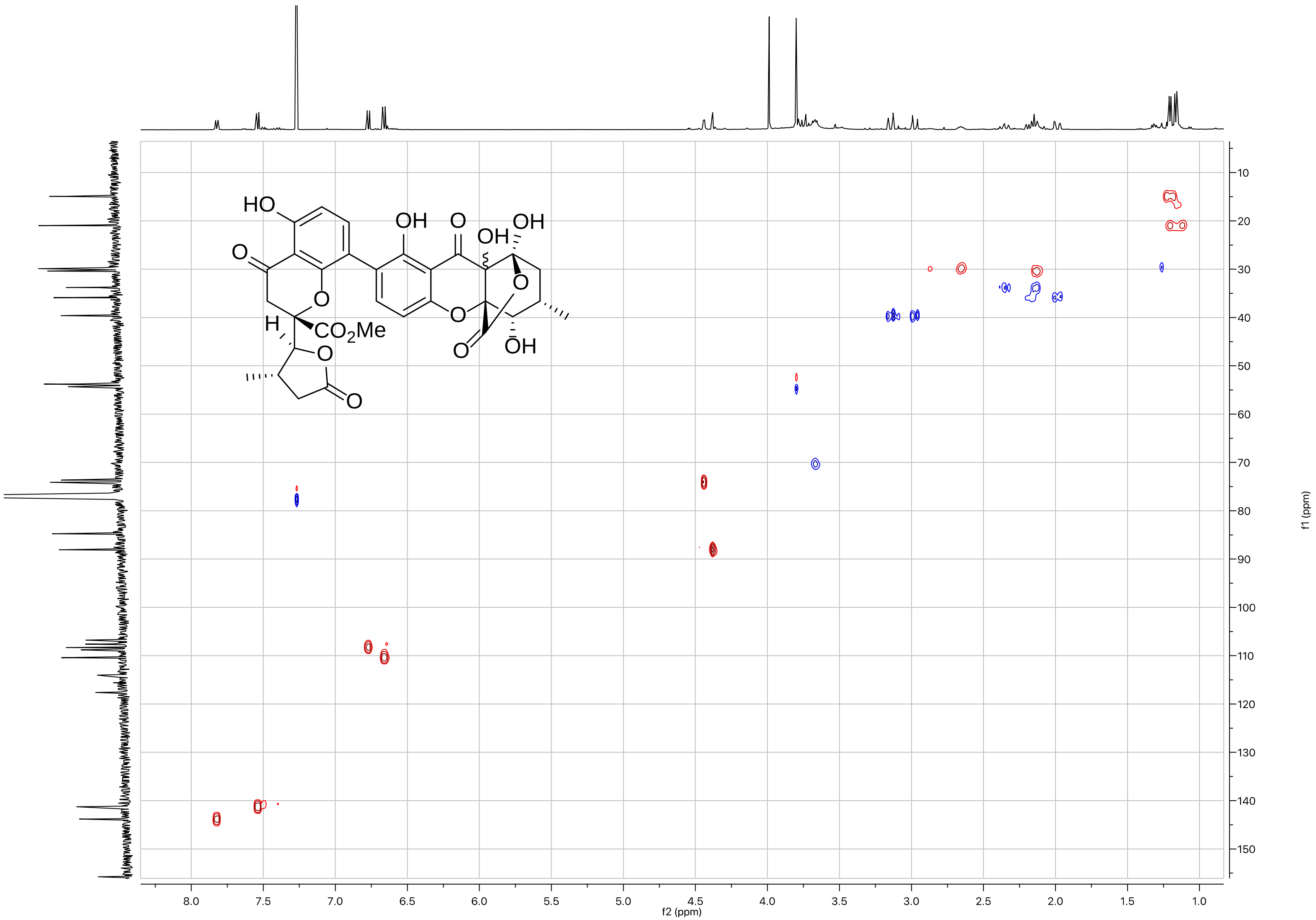

Figure S3. HSQC spectrum of pseudopalawanone A (**1**).

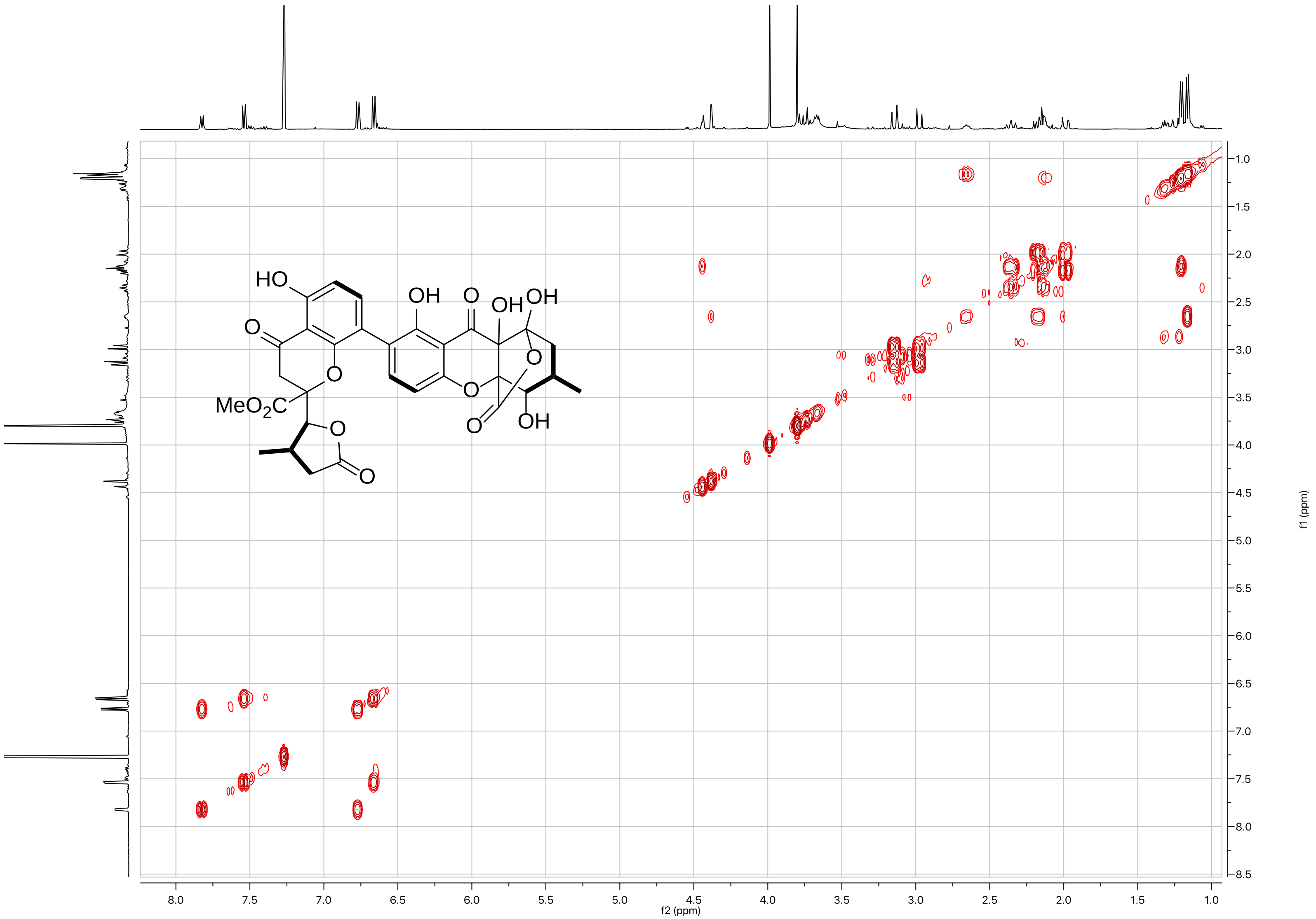

Figure S4. COSY spectrum of pseudopalawanone A (**1**).

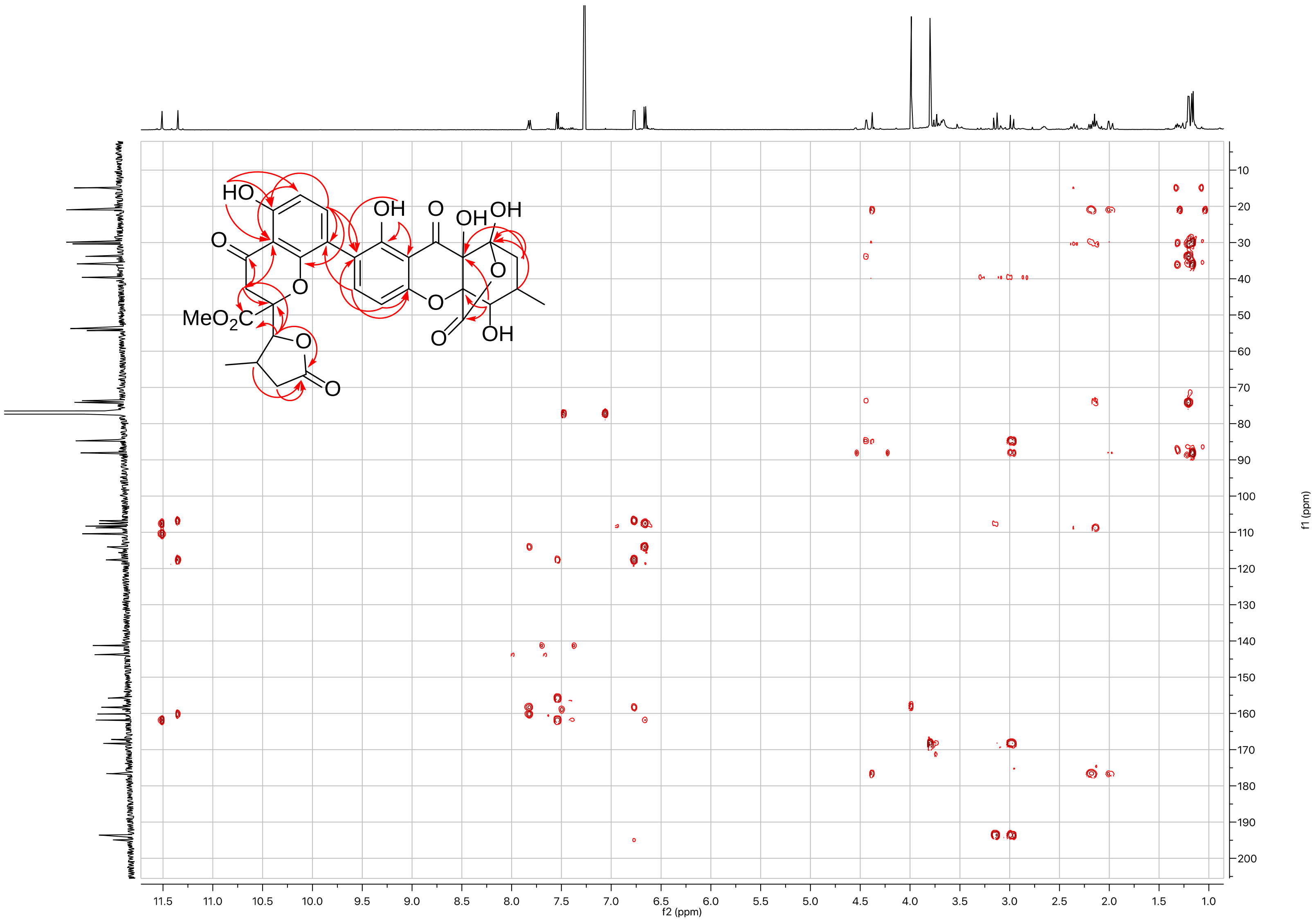

Figure S5. HMBC spectrum of pseudopalawanone A (**1**).

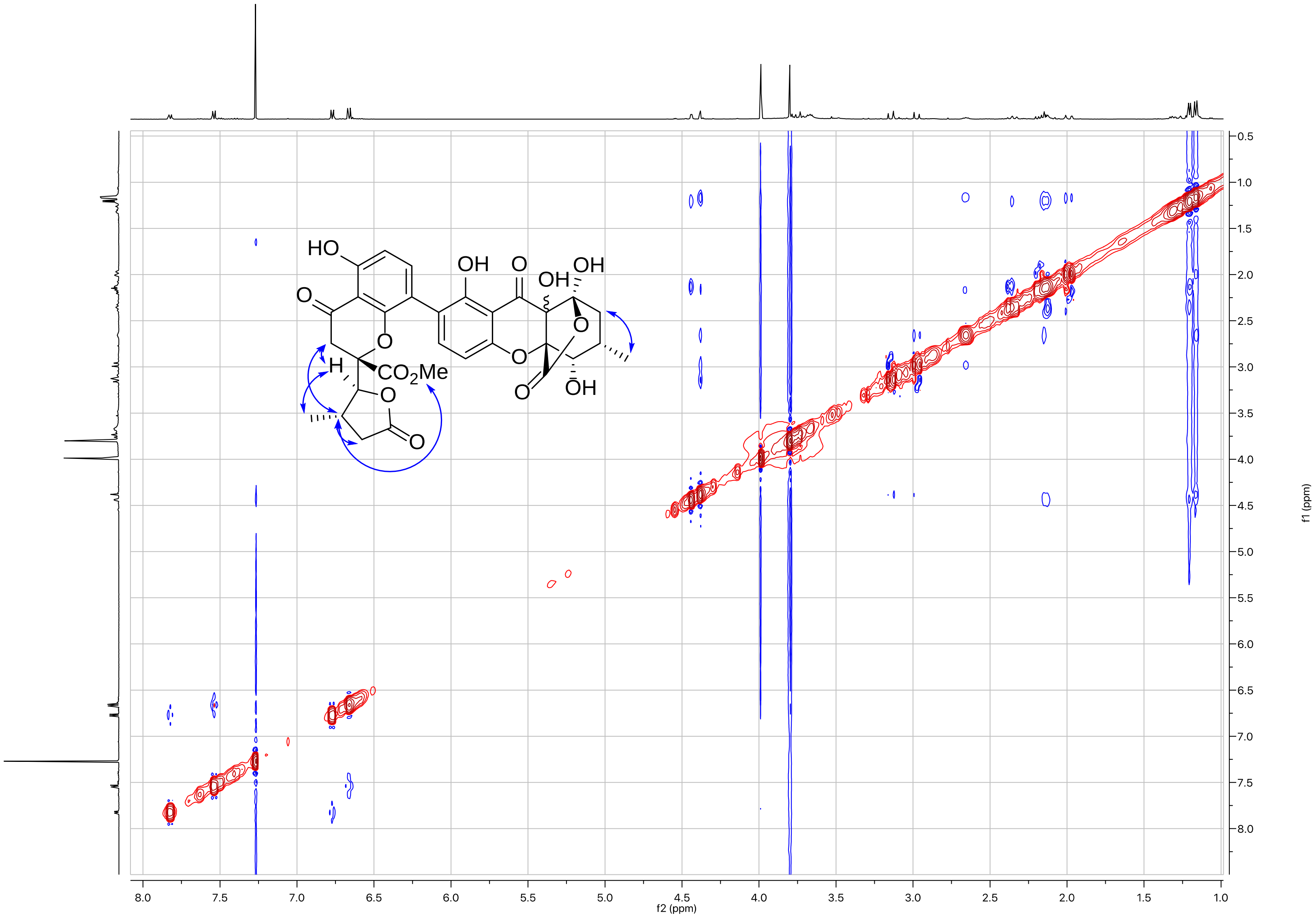

Figure S6. ROESY spectrum of pseudopalawanone A (**1**).

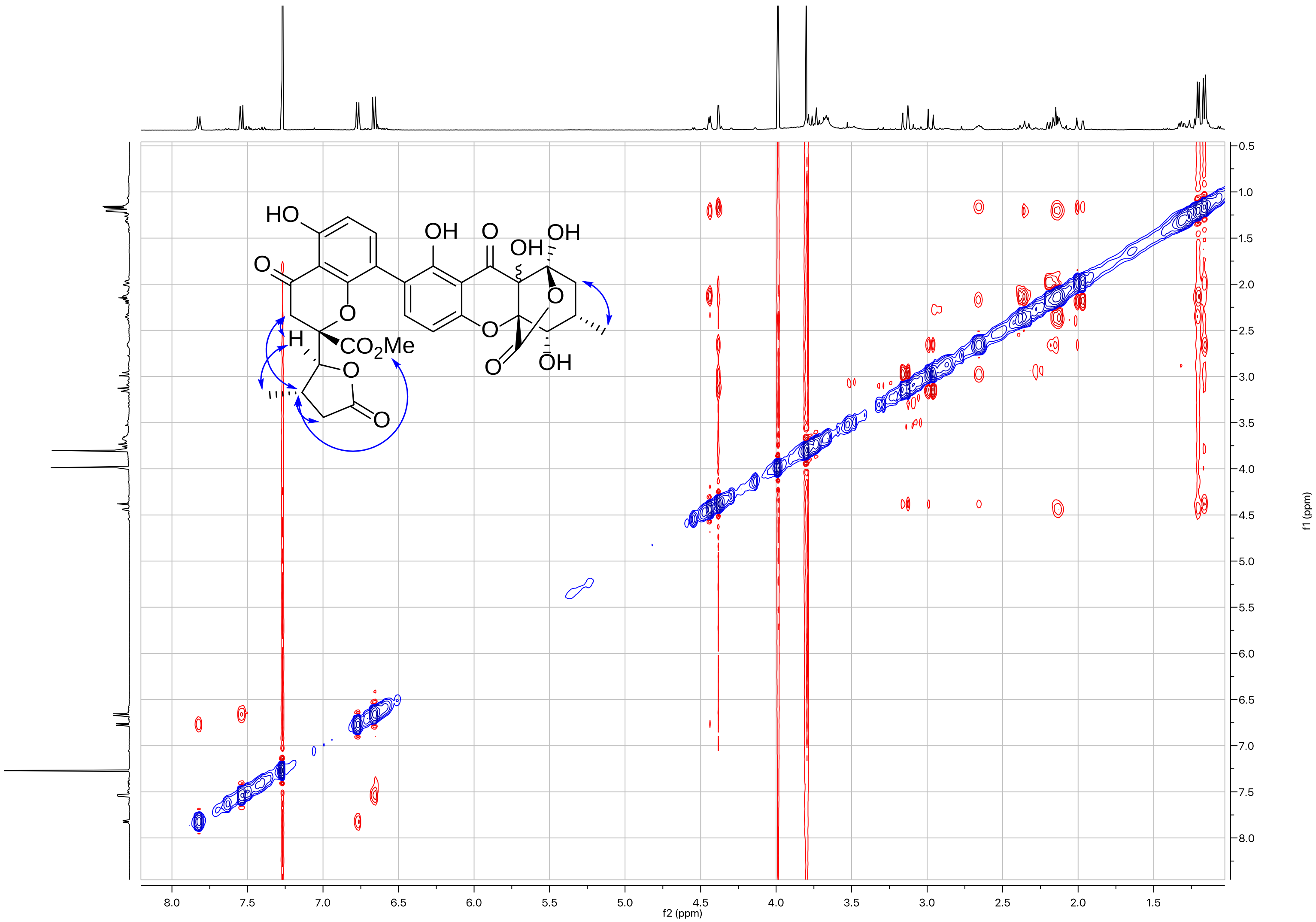

Figure S7. NOESY spectrum of pseudopalawanone A (**1**).

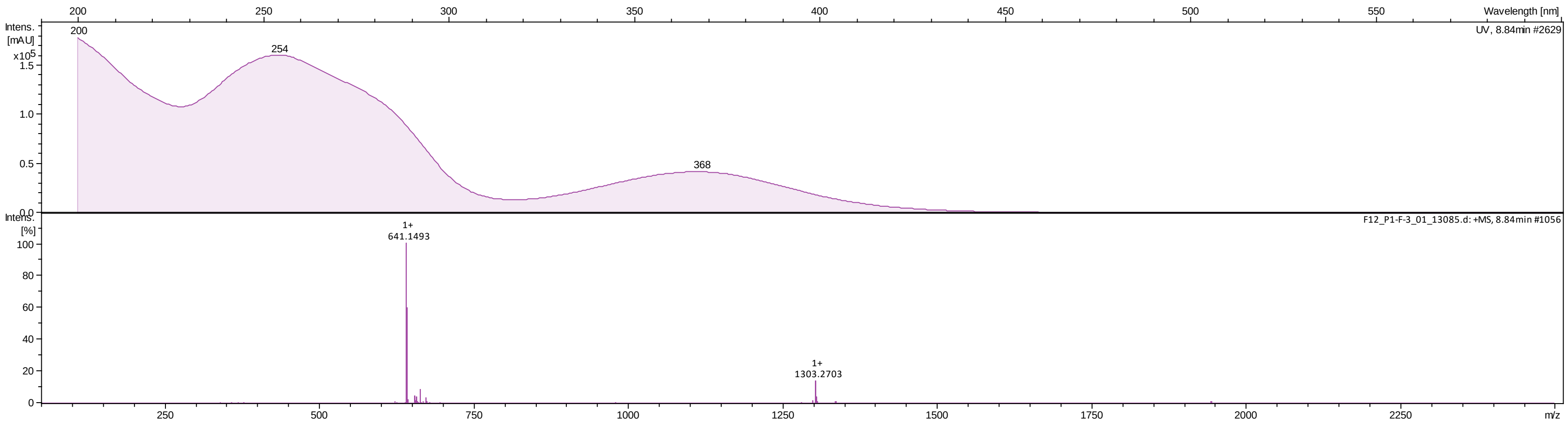

S8. HR-ESIMS spectrum of pseudopalawanone (**1**).

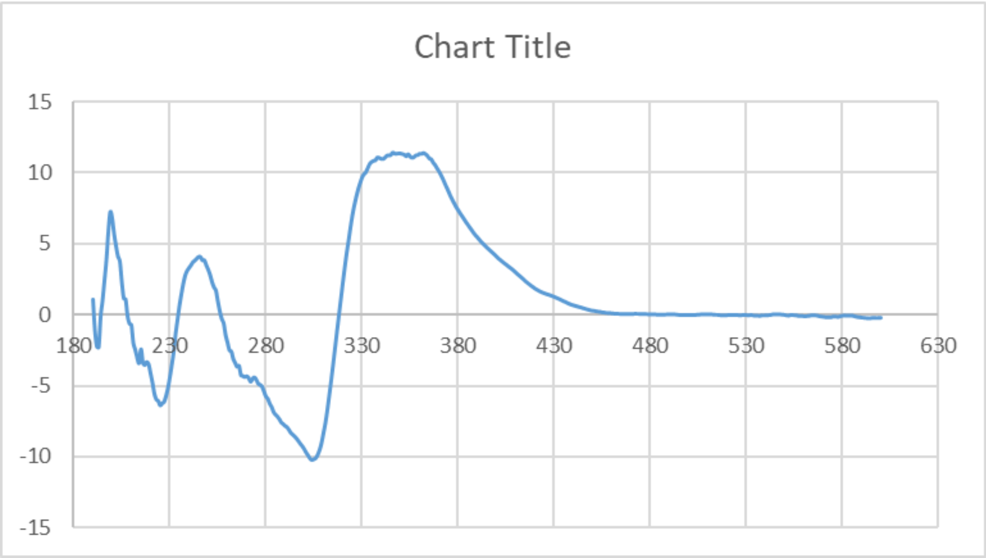

S9. CD spectrum (MeOH) of pseudopalawanone (**1**).
